# Identification of a seven-long non-coding RNA signature associated with Jab1/CSN5 in predicting hepatocellular carcinoma

**DOI:** 10.1038/s41420-021-00560-7

**Published:** 2021-07-10

**Authors:** Weijie Ma, Ye Yao, Gang Xu, Xiaoling Wu, Jinghua Li, Ganggang Wang, Xi Chen, Kunlei Wang, Yirang Chen, Yonghua Guo, Yongsheng Li, Deli Tan, Hui Guo, Zhisu Liu, Yufeng Yuan, Francois X. Claret

**Affiliations:** 1 Department of Hepatobiliary and Pancreatic Surgery, Zhongnan Hospital of Wuhan University, Wuhan, China; 2 Department of Systems Biology, The University of Texas - MD Anderson Cancer Center, Houston, TX USA; 3 Key Laboratory of Tropical Translational Medicine of Ministry of Education, Hainan Medical University, Haikou, 571199 China; 4 Department of Oncology, The First Affiliated Hospital, College of Medicine of Xi’an Jiaotong University, Xi’an, China

**Keywords:** Cancer epigenetics, Cancer epigenetics

## Abstract

Hepatocellular carcinoma (HCC) is a leading cause of cancer death worldwide, accounting for over 700,000 deaths each year. The lack of predictive and prognostic biomarkers for HCC, with effective therapy, remains a significant challenge for HCC management. Long non-coding RNAs (lncRNAs) play a key role in tumorigenesis and have clinical value as potential biomarkers in the early diagnosis and prediction of HCC. Jun activation domain-binding protein 1 (Jab1, also known as COP9 signalosome subunit 5, CSN5) is a potential oncogene that plays a critical role in the occurrence of HCC. Here, we performed a comprehensive analysis for Jab1/CSN5-associated lncRNAs to predict the prognosis of HCC. The differentially expressed (DE) lncRNAs between in HCC were analyzed based on the TCGA RNA-seq data. We detected 1031 upregulated lncRNAs in 371 HCC tissues and identified a seven-lncRNA signature strongly correlated with Jab1/CSN5 (SNHG6, CTD3065J16.9, LINC01604, CTD3025N20.3, KB-1460A1.5, RP13-582O9.7, and RP11-29520.2). We further evaluated the prognostic significance of these lncRNAs by GEPIA (http://gepia.cancer-pku.cn/). The expression data in 364 liver tumors indicated that this seven-lncRNA signature could better predict worse survival in HCC patients. Moreover, 35 clinical HCC samples were evaluated to assess the validity and reproducibility of the bioinformatic analysis. We found that the targeted lncRNAs were upregulated, with a strong association with Jab1/CSN5 and prognostic value in HCC. Functional enrichment analysis by Gene Ontology (GO) showed that these seven prognostic lncRNAs exhibit oncogenic properties and are associated with prominent hallmarks of cancer. Overall, our findings demonstrate the clinical implication of Jab1/CSN5 with the seven‐lncRNAs in predicting survival for patients with HCC.

## Highlights

- Seven-lncRNA signature associated with Jab1/CSN5 in HCC.
- Prognostic value of seven lncRNAs in HCC.
- Functional analysis of seven lncRNAs.

## Introduction

Hepatocellular carcinoma (HCC) is the second leading cause of death related to cancer among males and the sixth among females, accounting for over 700,000 deaths each year worldwide [1]. Prevention, early detection, and treatment improve cancer outcomes; however, the incidence of cancer mortality continues to rise in most HCC patients with a 5-year survival rate is still below 50% [2]. The level of exposure to environmental and infectious risk factors with early detection are the leading causes of the variation seen in liver cancer incidence and mortality [3]. Only 10–20% of HCC tumors can be removed by surgery, and the cumulative 5-year recurrence rate of patients who undergo curative hepatectomy averages 70% [4, 5]. Therefore, identifying novel biomarkers for predicting survival in HCC patients and efficient therapeutic targets is urgently needed.

Long non-coding RNAs (lncRNAs) are transcripts over 200 nucleotides in length with no protein-coding function, which are transcribed by RNA polymerase II similar to mRNAs [6]. Several studies have recently demonstrated the role of lncRNAs in diverse biological processes, including the regulation of epigenetic inheritance, transcriptional and post-transcriptional levels, imprinting, apoptosis, and drug resistance, and tumorigenesis via directly or indirectly regulating related PCGs (protein-coding genes) [6–10]. Most importantly, increasing evidence suggests that dysregulated lncRNAs play a crucial role in tumor initiation and progression [11, 12]. Several lncRNAs, including HOTAIR, HULC, and HOTTIP, are directly involved in tumorigenesis and metastasis in HCC [13–15].

Jun activation domain-binding protein 1 (Jab1, also known as COP9 signalosome subunit 5, CSN5/COPS5) is the fifth subunit of the COP9/signalosome complex (CSN5/COPS5) [16–20]. Recent findings suggest that Jab1/CSN5 is a potential oncogene that controls cell cycle regulation, cell proliferation, and tumor progression. Jab1/CSN5 is highly expressed in HCC tissues and acts as a new regulator of the p57 tumor suppressor gene by promoting its degradation and contributing to cell proliferation and tumor progression [21]. Thus, it is necessary to elucidate further the Jab1/CSN5-p57 axis role as a possible target therapy for HCC treatment. Moreover, recent studies showed that in the orthotopic transplant mouse models of human HCC, the systemic RNA interference targeting Jab1/CSN5 has strong potency without significant toxicity and suggested its clinical application in treating HCC disease [21, 22].

Both lncRNAs and Jab1/CSN5 are essential epigenetic regulators in cancer. However, the interactions between lncRNAs and Jab1/CSN5 have not been investigated. In this study, we applied a systematic identification of a lncRNA signature associated with Jab1/CSN5 involved in the growth and progression of HCC aiming to find Jab1/CSN5 related lncRNAs and evaluate their prognostic value in HCC. We generated a prognostic model and validated it to predict the prognostic lncRNA signature in HCC patients through bioinformatics analyses. We identified seven-lncRNA signature from The Cancer Genome Atlas (TCGA) LIHC dataset with clinical validations and determined its prognostic value was independent of clinical factors. For HCC, very few lncRNA signatures are discovered and developed for HCC prognosis prediction. Overall, our study identified an lncRNA signature that could predict HCC patients’ survival and could be used as a prognostic biomarker.

## Results

### Identification of differential expressed lncRNAs associated with CSN5 in LIHC cohort

**Fig. 1:**
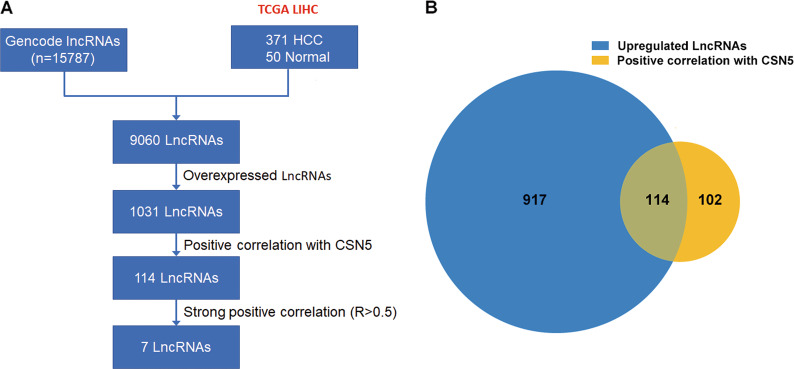
The flowchart for the current study. **A** The flowchart for the current study. **B** Venn diagram indicating the overlap between overexpressed lncRNAs and those positively correlated with CSN5. The overlap part indicated that 114 upregulated lncRNAs were positively correlated with CSN5.

**Fig. 2:**
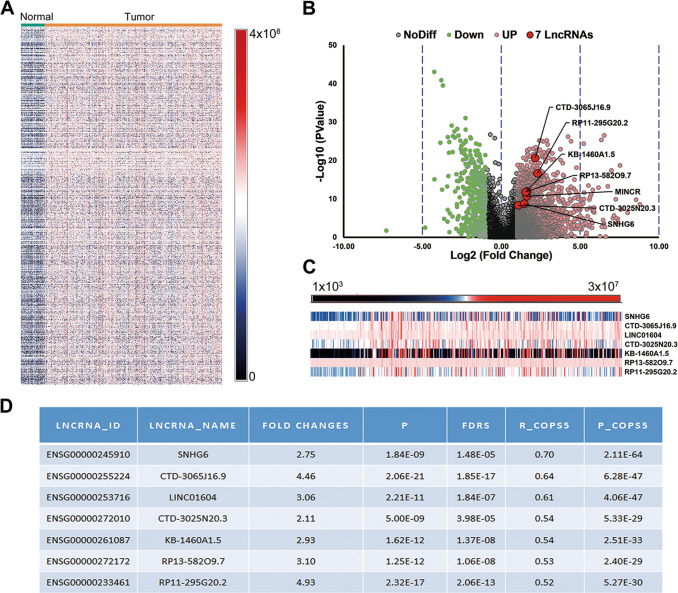
Differentially expressed lncRNAs in HCC from the TCGA database. **A** Heatmap of 1031 upregulated lncRNAs in 371 HCC tissues compared with 50 normal tissues **B** Volcano plot showed the distribution of the seven-lncRNA differential expression, which positively correlated with CSN5 co-expression between HCC and adjacent normal tissues (No Diff no difference, UP upregulation, Down downregulation). **C** Heatmap for the seven-lncRNA expression profiles. **D** Table presented the relative fold changes of lncRNAs in HCC, compared to normal samples, and the correlation coefficients with CSN5 levels.

The flowchart of this study is shown in Fig. 1A. A total of 1031 upregulated lncRNAs were detected in 371 HCC tissues and 50 normal tissues with HCC in the liver hepatocellular carcinoma (LIHC) cohort from the TCGA database. Among the 1031 upregulated lncRNAs, 114 lncRNAs had a positive correlation with CSN5 (*R* > 0.3, *P* < 0.05) (Fig. 1B). Next, we performed Spearman analysis by the threshold of *R* > 0.5, *P* < 0.05, to identify a stronger correlation between lncRNAs and Jab1/CSN5. We screened out seven targeted lncRNAs following the method’s criteria among these 1031 lncRNAs (heatmap shown in Fig. 2A). The volcano plot showed all the differentially expressed lncRNAs (log2|fold change|>1 and *P* < 0.05), among which the selected seven lncRNAs (SNH6,43 CTD3065J16.9, LINC01604, CTD3025N20.3, KB-1460A1.5, RP13-582O9.7, and 44 RP11-29520.2) were all significantly upregulated in HCC tissues, compared with normal control (Fig. 2B). The heatmap displayed the expression profiles of the seven lncRNAs in HCC samples and normal tissues (Fig. 2C). All seven lncRNAs were significantly upregulated in HCC compared with normal control (*P* < 0.001, FDR < 0.001). Besides, they all show a strong correlation (*R* > 0.5) with the Jab1/CSN5 levels (Fig. 2D).

### Prognostic value detection

**Fig. 3:**
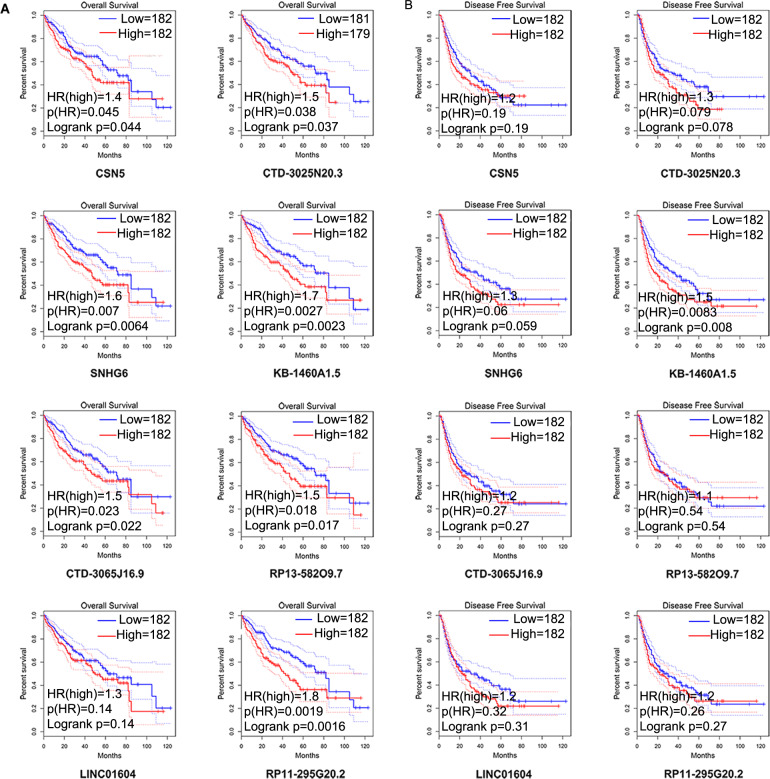
The prognostic assessment of seven lncRNAs from TCGA. **A** Kaplan–Meier survival curve of overall survival (OS) for the seven lncRNAs (with 95% confidence interval). **B** Kaplan–Meier survival curve of disease-free survival (DFS) for the seven-lncRNA signature.

Next, we applied GEPIA to perform the survival plots of the seven-lncRNAs and Jab1/CSN5 from the LIHC cohort (364 tumors) from the TCGA (Fig. 3). The high level of Jab1/CSN5 expression level predicted the worse overall survival. Intriguingly, most of the seven lncRNAs had a significant prognostic value on overall survival except LINC0164 (Fig. 3A). As to disease-free survival, only the level of KB-1460A1.5 was significant. The higher levels of SNHG6 and CTD3025N20.3 had a strong tendency to indicate the worse disease-free survival in HCC patients (Fig. 3B).

### Seven lncRNAs were upregulated in HCC tissue specimens and correlated with poor outcome

**Fig. 4:**
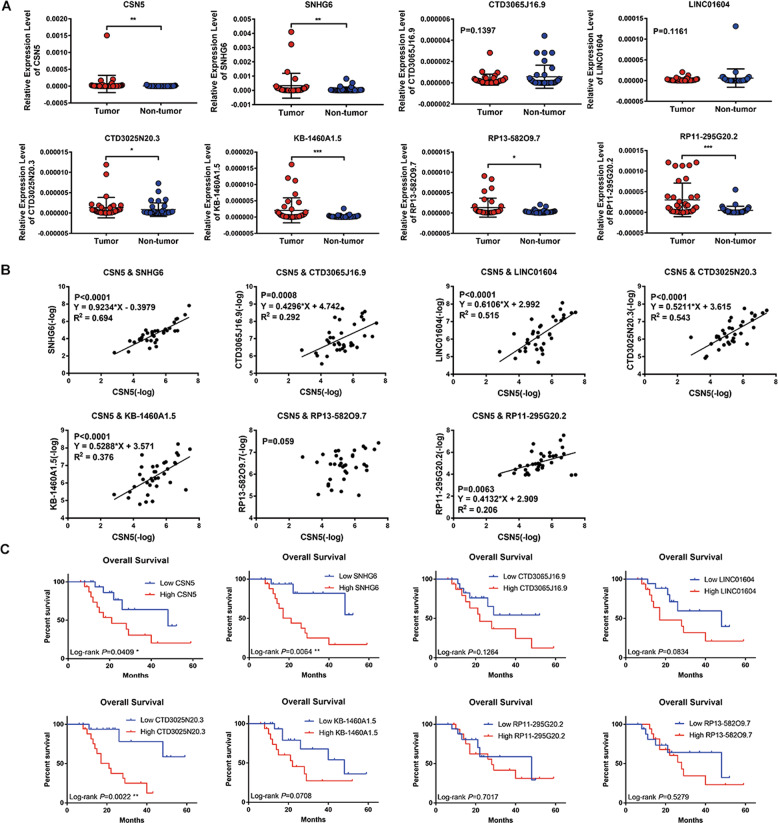
The relative expression level of CSN5 and seven lncRNAs from the Zhongnan cohort. **A** The CSN5 and seven lncRNAs expression levels were significantly higher than in adjacent non-tumor liver tissues. *P < 0.05, **P < 0.01. **B** The linear regression plots between the relative expression level in each of the CSN5 and seven-lncRNAs. The relative expression levels were normalized by the value of −log. **C** The prognostic value of CSN5 and seven lncRNAs of overall survival (OS) among the patients. *P < 0.05, **P < 0.01.

By testing and analyzing the relative expression level of seven-lncRNA signature and Jab1/CSN5 in 35 paired HCC tissues and adjacent normal hepatic tissues collecting from Zhongnan Hospital (Fig. 4), we found that Jab1/CSN5 was remarkably overexpressed in HCC compared with adjacent normal tissues (*P* < 0.01), as well as SNGH6, CTD3025N20.3, KB-1460A1.5, RP13-582O9.7, and RP11-29520.2 (Fig. 4A). However, we did not find a significant differential expression (DE) of CTD3065J16.9 and LINC01604 in clinical HCC samples. Besides, we found a strong correlation of this lncRNA signature with Jab1/CSN5 from our cohort, except RP13-582O9.7 (*P* = 0.059) (Fig. 4B). We divided these 35 HCC patients into low and high expression groups according to the median level of expression. We then tested the correlation between the relative expression of seven-lncRNA and CSN5 and the overall survival of HCC patients through Kaplan–Meier analysis and log-rank test. As Fig. 4C shows, HCC patients with higher Jab1/CSN5, SNHG6, or CTD3025N20.3 expression significantly predicted shorter overall survival. Univariate analysis indicated that the difference in the expression levels of CSN5, SNHG6, and CTD3025N20.3 was significantly related to the overall survival of HCC patients (*P* < 0.05, Table 1). The level of CTD3025N20.3 was an independent favorable prognostic factor of HCC, as demonstrated by multivariate analysis with the Cox as the proportional hazards model (*P* < 0.05, Table 1).

**Table 1:** Prognostic factors in Cox proportional hazards model.

Factors (*n* = 35)	Univariate analysis		Multivariate analysis	
	HR (95% CI)	*P*	HR (95% CI)	*P*
Gender (male/female)	0.319 (0.088–1.155)	0.082		
Age (⩽60/>60)	1.699 (0.635–4.549)	0.291		
Differentiation (low/high or moderate)	3.687 (1.245–10.922)	0.019		
TNM (III∼IV/I∼II)	0.605 (0.219–1.674)	0.334		
CSN5 (high/low)	2.889 (0.995–8.393)	0.041		
SNHG6 (high/low)	4.925 (1.387–17.484)	0.014		
CTD3065J16.9 (high/low)	2.159 (0.783–5.952)	0.137		
LINC01604 (high/low)	2.401 (0.862–6.688)	0.094		
CTD3025N20.3 (high/low)	7.311 (1.644–32.518)	0.009	7.311 (1.644–32.518)	0.009
KB-1460A1.5 (high/low)	2.503 (0.893–7.018)	0.081		
RP13-582O9.7 (high/low)	1.383 (0.502–3.814)	0.531		
RP11-295G20.2 (high/low))	1.221 (0.438–3.403)	0.703		

*TNM* tumor-node-metastasis. *P* < 0.05 was considered statistically significant.

### Association between the seven-lncRNA signature and clinic-pathologic characteristics of HCC

**Fig. 5:**
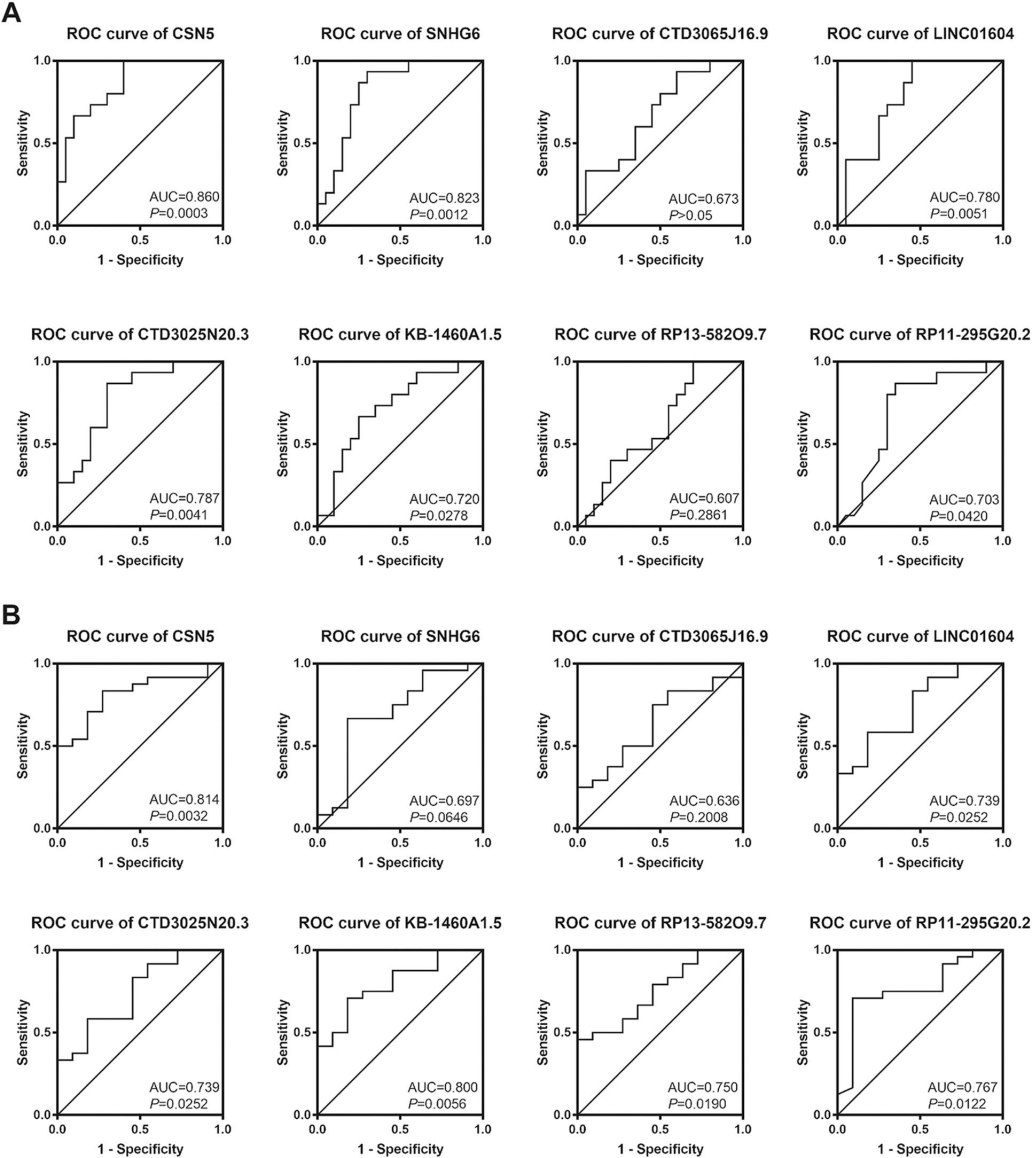
ROC curves of CSN5 and seven lncRNAs expression. **A** The relative expression levels were proven to be a valuable parameter in the differentiation of HCC. **B** The predictive value of CSN5 and seven lncRNAs for the TNM stage of HCC.

Next, we assessed the association between the seven-lncRNA signature and CSN5 with the clinical characteristics of 35 HCC patients (Table S2) (*P* < 0.05). The expression level of the seven-lncRNA and Jab1/CSN5 were obtained from quantitative real-time PCR (qRT-PCR). Furthermore, we performed Student’s *T*-tests and found that the Jab1/CSN5 level was closely related to tumor differentiation, TNM stage, and HCC patients’ mortality. We also found a significant correlation between the seven lncRNAs and clinical characteristics of HCC patients. Expression levels of SNHG6, CTD3025N20.3, and KB-1460A1.5 were associated with the gender of patients. Except for RP13-582O9.7, the rest of the six-lncRNA cluster was correlated with tumor differentiation and Jab1/CSN5 level of the HCC patients (see details in Table S2). Overexpressed levels of SNHG6, CTD3025N20.3, KB-1460A1.5, RP13-582O9.7, and RP11-295G20.2 could predict a more advanced TNM stage. We utilized receiver operating characteristic (ROC) curves to analyze the diagnostic accuracy expressed in the degree of differentiation and TNM stages (Fig. 5). The relative expression levels of the seven-lncRNAs were a valuable parameter in the differentiation and TNM staging of HCC.

### Functional enrichment analysis

**Fig. 6:**
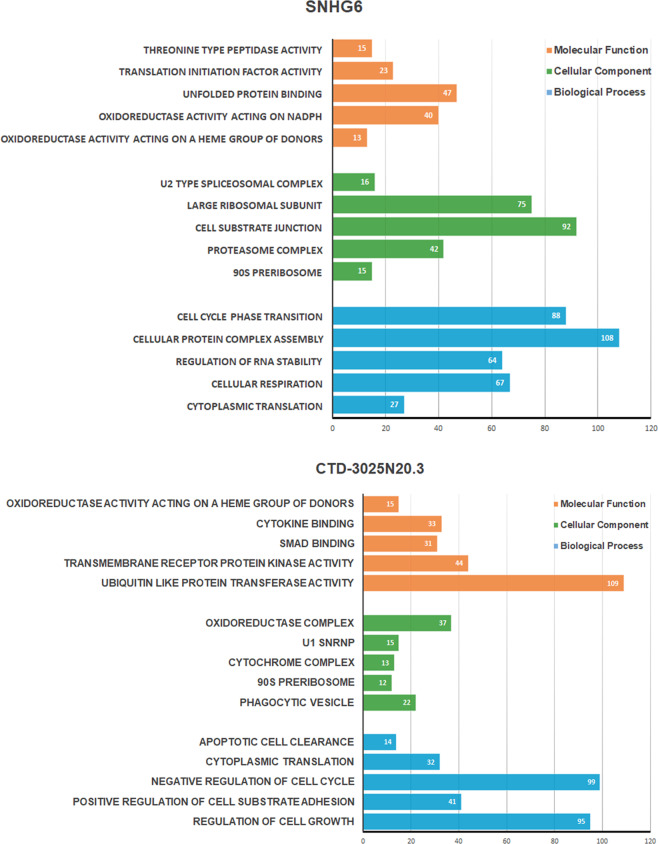
GO functional enrichment analysis of lncRNAs. Functional enrichment for SNHG6 (up) and CTD-3025N20.3 (down) were partly presented.

Functional enrichment analysis by the DAVID web annotation tool was performed to explore the potential biological functions of our seven-lncRNA signature. As indicated by Gene Ontology (GO) enrichment analysis, the co-expressed PCGs were highly enriched in a crowd of GO terms. Figure 6 and Supplementary Fig. S1 partly showed that the lncRNAs-related PCGs were mostly involved in the cell cycle phase transition, cell apoptosis, and regulation of cell growth, related to Jab1/CSN5 biological function (Fig. 6). Based on our analyses, we proposed that our seven-lncRNA signature might regulate the tumorigenesis of HCC.

## Discussion

Jab1/CSN5 is implicated in regulating key cellular biological processes, including cellular proliferation, cell cycle control, apoptosis, regulation of instability of the genome, and DNA damage response [16–18, 23]. Jab1/CSN5 is overactivated in different cancer types, including nasopharyngeal carcinoma, breast cancer, pancreatic, and lung cancers, and was related mechanistically to promote tumor progression and poor prognosis [24–28]. Hsu et al. [29] demonstrated that Jab1/CSN5 was upregulated in HCC and suppressed Jab1/CSN5 induced by peroxisome proliferator-activated receptor-gamma (PPARγ) ligands cause the induction of cell apoptosis and the inhibition of cell cycle progression, which impair the proliferation of HCC. Our previous work also found overexpression of Jab1/CSN5 significantly predicted the poor outcome of HCC patients, which mediated p57 degradation leading to hepatocarcinogenesis [21]. Recently, peptidomimetic antagonists for Jab1/CSN5 had been exploited since the C-terminal domain of one molecule docks in another one’s active site [30]. Schlierf et al. [31] recently reported an effective, specific, and orally available Jab1/COPS5 inhibitor CSN5i-3, which trapped cullin-RING E3 ubiquitin ligases (CRLs) in the neddylated state, inactivating a subset of CRLs by the degradation of their substrate recognition module. It was demonstrated that CSN5i-3 significantly inhibited tumor cell proliferation and reduced tumor growth [31]. These results shed light on the potential of Jab1/CSN5 as a therapeutic target of tumor treatment.

LncRNAs play different roles in biological processes, including the interaction with one or more protein partners [32]. Specific lncRNAs can accumulate to the transcription start site and interact with proteins to regulate the gene expression in *cis* [33]. Others play the role of molecular decoys to prevent specific transcription factors from interacting with DNA by intramolecular binding [34]. Furthermore, they could combine with antisense mRNAs and regulate post-transcriptional mechanisms or serve as a scaffold to assemble macromolecular complexes [35, 36].

In this study, we applied comprehensive bioinformatics analysis to identify the differential expressed lncRNAs positively related to Jab1/CSN5 and with poor survival of HCC. We identified seven targeted lncRNA signature, SNHG6, CTD3065J16.9, LINC01604, CTD3025N20.3, KB-1460A1.5, RP13-582O9.7, and RP11-295G20.2. As far as we know, the role of this seven-lncRNA in connection with Jab1/CSN5 has not been studied. SNHG6 (small nucleolar RNA host gene 6), located on chromosome 8q13, was identified as an oncogene in several cancers, including gastric cancer, glioma, HCC, and osteosarcoma [37–40]. Our previous study reported that overexpressed SNHG6 could predict poor prognosis among HCC patients [39]. SNHG6 facilitated cellular proliferation, inhibits cell apoptosis, and promotes cell cycle progression. Moreover, SNHG6 can act as a ceRNA in the regulation of ZEB1 expression by competitively binding miR-101-3p and improve HCC tumorigenesis by binding UPF1 (Up-frameshift protein 1), leading to TGF-b/Smad pathway activation [39]. SNHG6 inhibited the expression of MAT1A protein, which was expressed in quiescent adult hepatocytes and upregulated the SAMe concentration in the liver [41], by the activation of the miR-1297/FUS (fused in sarcoma) pathway to modify the nucleocytoplasmic shuttling of MAT1A mRNA and promoted the expression of MAT2A by inhibiting the direct binding of miR-1297 to the MAT2A 3′UTR [42]. These findings demonstrated the role of SNHG6 in promoting HCC. Intriguingly, according to our findings, SNHG6 was significantly related to Jab1/CSN5, thus suggesting that SNHG6 could regulate Jab1/CSN5 in the tumorigenesis of HCC.

Next, we conducted a comprehensive analysis of the lncRNA expression profile and related clinical information of 364 HCC patients from TCGA. The prognostic values were detected through a log-rank test for the overall survival and disease-free survival from the TCGA database. We analyzed 35 clinical HCC samples for Jab1/CSN5 and the seven-lncRNA expression levels by qPCR to verify the reproductivity of the results from the bioinformatic analysis. In our HCC cohort, CTD3065J16.9 and LINC01604 failed the present their upregulation, RP13-582O9.7 did not show a significant correlation with Jab1/CSN5 (*P* = 0.059) due to the small sample size. Vital pathologic characteristics, like differentiation, were related to SNHG6, CTD3065J16.9, LINC01604, CTD3025N20.3, KB-1460A1.5, and RP11-29520.2. TNM stage was related to SNHG6, LINC01604, CTD3025N20.3, KB-1460A1.5, RP13-582O9.7, and RP11-29520.2. Jab1/CSN5, SNHG6, or CTD3025N20.3 levels significantly indicate shorter overall survival.

We further evaluate the biological functions by functional enrichment analyses of GO for lncRNA-related PCGs and found enrichment in essential cellular activities, cytoplasmic translation, oxidoreductase activity, and unfolded protein binding. The functional enrichment analyses indicate that this seven-lncRNA signature could play critical roles in cellular function and protein interactions. Intriguingly, these seven-lncRNAs are also involved in cell cycle phase transition, regulation of cell growth, and cell proliferation which are critical pathways controlled by Jab1/CSN5. However, further studies are required to validate this seven-lncRNA signature with the exact interaction mechanisms between them and Jab1/CSN5 and their biological functions, validate the lncRNA signature’s predictive value in a prospective cohort of HCC patients. To summarize, integrative HCC lncRNA expression analysis based on the TCGA database with qPCR validation in the HCC cohort identified seven lncRNAs signature related to Jab1/CSN5. The signature has the potential to serve as a prognostic biomarker to improve prediction for HCC patients, and pathways associated with it might enable the development of novel therapies for HCC treatment.

## Materials and methods

### TCGA dataset of lncRNAs expression

An RNA sequencing (RNA-Seq) dataset of the LIHC cohort was downloaded from the TCGA database (https://cancergenome.nih.gov). The present study included 371 HCC tumor tissues with gene expression profiles and 50 normal tissues from 50 patients with HCC. According to GENCODE’s annotations, lncRNAs, and PCG were identified from the expression matrix (http://www.gencodegenes.org).

### Screening of differentially expressed lncRNAs

We used the edgeR package in R software to identify the DE of lncRNA between 371 HCC tissues and 50 normal tissues [43]. We set the threshold of lncRNA differential expression to log fold change (FC) > 2 and false discovery rate (FDR) < 0.01. Then, we applied the threshold as *R* > 0.5, *P* < 0.05, to screen out the lncRNAs, which were positively correlated with Jab1/CSN5. Wilcox rank-sum test and Spearman rank-order correlation were executed. Then, we tested the prognostic value of these lncRNAs by GEPIA (Gene Expression Profiling Interactive Analysis) (http://gepia.cancer-pku.cn/) [44].

### Ethical statement

This study was conducted according to the recommendations of the Ethics Committee of Zhongnan Hospital of Wuhan University, and the written informed consent of all subjects was obtained. All subjects signed an informed consent based on the “Declaration of Helsinki”. The protocol received the approval of the Ethics Committee of Zhongnan Hospital of Wuhan University (No. 2018013).

### Collection of clinical HCC data

We collected paired clinical tissue specimens (tumor and neighboring normal/healthy tissues) from 35 HCC patients who underwent surgery during 2015–2018 (Zhongnan Hospital of Wuhan University, China) and obtained their consent to participate in the study. No radiotherapy nor chemotherapy was performed before surgery. An experienced pathologist confirmed all tissue specimens histologically. Fresh tissue samples collection and the definition of tumor staging have been described earlier [45].

### qRT-PCR analysis

Total RNA from tissues and cells was isolated using TRIzol reagent (Invitrogen, CA, USA). The concentration and purity of RNA were quantified by NanoDrop ND2000 (Thermo, CA, USA). One microgram RNA was transcribed reversely, as previously described [45]. The qRT-PCR reaction was performed on CFX96TM Real-Time System (Bio-Rad, CA, USA). The reactions began at 95 °C for 5 min, then 40 cycles of 95 °C for 30 s, followed by 60 °C for 30 s, and ending with 72 °C for 30 s. Eighteen seconds served as an internal control. All the synthesized primer sequences are listed in Table S1. To calculate the relative gene expression, we used the comparative cycle threshold (Ct) method (2 − ΔCt).

### Functional enrichment analysis

Biological processes involved in the lncRNA signature were predicted using functional enrichment analysis of GO in Database for Annotation, Visualization, and Integrated Discovery (DAVID) Bioinformatics Resources 6.8 (https://david.ncifcrf.gov/) online annotation tool [46, 47]. The criteria were set an enrichment score >2 and *P* value <0.05 for significant enriched GO terms.

### Statistical analysis

The continuous variable was represented as the mean ± standard deviation (SD). We performed the *t*-test or Mann–Whitney *U-*test to assess the differences in continuous variables. For categorical variables, the Chi-squared test or Fisher’s exact test was performed to find evidence between the different categories. We used the Kaplan–Meier test to evaluate the lncRNA level’s impact to predict the overall survival of HCC patients, and for the analysis of the statistical differences between different groups, the log-rank test was used. Multivariate analysis was performed to set the proportional hazards model, the Cox with *P* < 0.05 as a standard to contain the variables in univariate analysis. We further designed a time-dependent ROC curve and Harrell consistency index (*C*-index) to evaluate the predictive capacity of the lncRNAs expression. The statistical analyses were performed by SPSS Statistics 24.0 (SPSS Inc., Chicago, IL, USA) GraphPad Prism 7.0 (GraphPad Software, La Jolla, CA, USA). *P* < 0.05 was defined as the criteria of statistical significance, which was assigned at *P < 0.05 or **P < 0.01.

## Supplementary information

Supplementary Figure Legends

Fig. S1

Table-S2

## Data Availability

The datasets generated and/or analyzed during the current study are available from the corresponding author on reasonable request.

## References

[1] Siegel RL, Miller KD, Jemal A (2020). Cancer statistics, 2020. CA Cancer J Clin.

[2] Global Burden of Disease Cancer C, Fitzmaurice C, Abate D, Abbasi N, Abbastabar H, Abd-Allah F, et al. Global, regional, and national cancer incidence, mortality, years of life lost, years lived with disability, and disability-adjusted life-years for 29 cancer groups, 1990 to 2017: a systematic analysis for the global burden of disease study. JAMA Oncol. 2019;12:1749–68. 10.1001/jamaoncol.2019.2996 PMC6777271 31560378

[3] Yang JD, Hainaut P, Gores GJ, Amadou A, Plymoth A, Roberts LR (2019). A global view of hepatocellular carcinoma: trends, risk, prevention and management. Nat Rev Gastroenterol Hepatol.

[4] Altekruse SF, McGlynn KA, Reichman ME (2009). Hepatocellular carcinoma incidence, mortality, and survival trends in the United States from 1975 to 2005. J Clin Oncol.

[5] Petrick JL, Florio AA, Loomba R, McGlynn KA (2020). Have incidence rates of liver cancer peaked in the United States?. Cancer.

[6] Mercer TR, Dinger ME, Mattick JS (2009). Long non-coding RNAs: insights into functions. Nat Rev Genet.

[7] Hangauer MJ, Vaughn IW, McManus MT (2013). Pervasive transcription of the human genome produces thousands of previously unidentified long intergenic noncoding RNAs. PLoS Genet.

[8] Cheetham SW, Gruhl F, Mattick JS, Dinger ME (2013). Long noncoding RNAs and the genetics of cancer. Br J Cancer.

[9] Consortium EP, Birney E, Stamatoyannopoulos JA, Dutta A, Guigo R, Gingeras TR (2007). Identification and analysis of functional elements in 1% of the human genome by the ENCODE pilot project. Nature.

[10] Fatica A, Bozzoni I (2014). Long non-coding RNAs: new players in cell differentiation and development. Nat Rev Genet.

[11] Schmitt AM, Chang HY (2016). Long noncoding RNAs in cancer pathways. Cancer Cell.

[12] Spizzo R, Almeida MI, Colombatti A, Calin GA (2012). Long non-coding RNAs and cancer: a new frontier of translational research?. Oncogene.

[13] Panzitt K, Tschernatsch MM, Guelly C, Moustafa T, Stradner M, Strohmaier HM (2007). Characterization of HULC, a novel gene with striking up-regulation in hepatocellular carcinoma, as noncoding RNA. Gastroenterology.

[14] Gupta RA, Shah N, Wang KC, Kim J, Horlings HM, Wong DJ (2010). Long non-coding RNA HOTAIR reprograms chromatin state to promote cancer metastasis. Nature.

[15] Quagliata L, Matter MS, Piscuoglio S, Arabi L, Ruiz C, Procino A (2014). Long noncoding RNA HOTTIP/HOXA13 expression is associated with disease progression and predicts outcome in hepatocellular carcinoma patients. Hepatology.

[16] Pan Y, Yang H, Claret FX (2014). Emerging roles of Jab1/CSN5 in DNA damage response, DNA repair, and cancer. Cancer Biol Ther.

[17] Shackleford TJ, Claret FX (2010). JAB1/CSN5: a new player in cell cycle control and cancer. Cell Div.

[18] Liu G, Claret FX, Zhou F, Pan Y (2018). Jab1/COPS5 as a novel biomarker for diagnosis, prognosis, therapy prediction and therapeutic tools for human cancer. Front Pharmacol.

[19] Seeger M, Kraft R, Ferrell K, Bech-Otschir D, Dumdey R, Schade R (1998). A novel protein complex involved in signal transduction possessing similarities to 26S proteasome subunits. FASEB J.

[20] Wei N, Tsuge T, Serino G, Dohmae N, Takio K, Matsui M (1998). The COP9 complex is conserved between plants and mammals and is related to the 26S proteasome regulatory complex. Curr Biol.

[21] Guo H, Jing L, Cheng Y, Atsaves V, Lv Y, Wu T (2016). Down-regulation of the cyclin-dependent kinase inhibitor p57 is mediated by Jab1/Csn5 in hepatocarcinogenesis. Hepatology.

[22] Lee YH, Judge AD, Seo D, Kitade M, Gomez-Quiroz LE, Ishikawa T (2011). Molecular targeting of CSN5 in human hepatocellular carcinoma: a mechanism of therapeutic response. Oncogene.

[23] Lee MH, Zhao R, Phan L, Yeung SC (2011). Roles of COP9 signalosome in cancer. Cell Cycle.

[24] Pan Y, Zhang Q, Tian L, Wang X, Fan X, Zhang H (2012). Jab1/CSN5 negatively regulates p27 and plays a role in the pathogenesis of nasopharyngeal carcinoma. Cancer Res.

[25] Kouvaraki MA, Korapati AL, Rassidakis GZ, Tian L, Zhang Q, Chiao P (2006). Potential role of Jun activation domain-binding protein 1 as a negative regulator of p27kip1 in pancreatic adenocarcinoma. Cancer Res.

[26] Kouvaraki MA, Rassidakis GZ, Tian L, Kumar R, Kittas C, Claret FX (2003). Jun activation domain-binding protein 1 expression in breast cancer inversely correlates with the cell cycle inhibitor p27(Kip1). Cancer Res.

[27] Osoegawa A, Yoshino I, Kometani T, Yamaguchi M, Kameyama T, Yohena T (2006). Overexpression of Jun activation domain-binding protein 1 in nonsmall cell lung cancer and its significance in p27 expression and clinical features. Cancer.

[28] Pan Y, Zhang Q, Atsaves V, Yang H, Claret FX (2013). Suppression of Jab1/CSN5 induces radio- and chemo-sensitivity in nasopharyngeal carcinoma through changes to the DNA damage and repair pathways. Oncogene.

[29] Hsu MC, Huang CC, Chang HC, Hu TH, Hung WC (2008). Overexpression of Jab1 in hepatocellular carcinoma and its inhibition by peroxisome proliferator-activated receptor{gamma} ligands in vitro and in vivo. Clin Cancer Res.

[30] Echalier A, Pan Y, Birol M, Tavernier N, Pintard L, Hoh F (2013). Insights into the regulation of the human COP9 signalosome catalytic subunit, CSN5/Jab1. Proc Natl Acad Sci USA.

[31] Schlierf A, Altmann E, Quancard J, Jefferson AB, Assenberg R, Renatus M (2016). Targeted inhibition of the COP9 signalosome for treatment of cancer. Nat Commun.

[32] Zhu J, Fu H, Wu Y, Zheng X (2013). Function of lncRNAs and approaches to lncRNA-protein interactions. Sci China Life Sci.

[33] Wang X, Arai S, Song X, Reichart D, Du K, Pascual G (2008). Induced ncRNAs allosterically modify RNA-binding proteins in cis to inhibit transcription. Nature.

[34] Geisler S, Coller J (2013). RNA in unexpected places: long non-coding RNA functions in diverse cellular contexts. Nat Rev Mol Cell Biol.

[35] Tripathi V, Ellis JD, Shen Z, Song DY, Pan Q, Watt AT (2010). The nuclear-retained noncoding RNA MALAT1 regulates alternative splicing by modulating SR splicing factor phosphorylation. Mol Cell.

[36] Carrieri C, Cimatti L, Biagioli M, Beugnet A, Zucchelli S, Fedele S (2012). Long non-coding antisense RNA controls Uchl1 translation through an embedded SINEB2 repeat. Nature.

[37] Yan K, Tian J, Shi W, Xia H, Zhu Y (2017). LncRNA SNHG6 is associated with poor prognosis of gastric cancer and promotes cell proliferation and EMT through epigenetically silencing p27 and sponging miR-101-3p. Cell Physiol Biochem.

[38] Cai G, Zhu Q, Yuan L, Lan Q (2018). LncRNA SNHG6 acts as a prognostic factor to regulate cell proliferation in glioma through targeting p21. Biomed Pharmacother.

[39] Chang L, Yuan Y, Li C, Guo T, Qi H, Xiao Y (2016). Upregulation of SNHG6 regulates ZEB1 expression by competitively binding miR-101-3p and interacting with UPF1 in hepatocellular carcinoma. Cancer Lett.

[40] Ruan J, Zheng L, Hu N, Guan G, Chen J, Zhou X (2018). Long noncoding RNA SNHG6 promotes osteosarcoma cell proliferation through regulating p21 and KLF2. Arch Biochem Biophys.

[41] Lu SC, Mato JM (2012). S-adenosylmethionine in liver health, injury, and cancer. Physiol Rev.

[42] Guo T, Wang H, Liu P, Xiao Y, Wu P, Wang Y (2018). SNHG6 acts as a genome-wide hypomethylation trigger via coupling of miR-1297-mediated S-adenosylmethionine-dependent positive feedback loops. Cancer Res.

[43] Chen Y, Lun ATL, Smyth GK. Differential Expression Analysis of Complex RNA-seq Experiments Using edgeR. In: Datta S., Nettleton D. (eds) Statistical Analysis of Next Generation Sequencing Data. Frontiers in Probability and the Statistical Sciences. Springer, Cham. 2014. 10.1007/978-3-319-07212-8_3.

[44] Tang Z, Li C, Kang B, Gao G, Li C, Zhang Z (2017). GEPIA: a web server for cancer and normal gene expression profiling and interactive analyses. Nucleic Acids Res.

[45] Ma W, Chen X, Wu X, Li J, Mei C, Jing W (2020). Long noncoding RNA SPRY4-IT1 promotes proliferation and metastasis of hepatocellular carcinoma via mediating TNF signaling pathway. J. Cell Physiol.

[46] Huang da W, Sherman BT, Lempicki RA (2009). Bioinformatics enrichment tools: paths toward the comprehensive functional analysis of large gene lists. Nucleic Acids Res.

[47] Huang da W, Sherman BT, Lempicki RA (2009). Systematic and integrative analysis of large gene lists using DAVID bioinformatics resources. Nat Protoc.

