# Supplementary material for: Identification of a seven-long non-coding RNA signature associated with Jab1/CSN5 in predicting hepatocellular carcinoma
Source: Cell Death Discov. 2021 Jul 10;7:178. doi: 10.1038/s41420-021-00560-7 (PMC8272716; doi:10.1038/s41420-021-00560-7)
Supplement: Supplementary file 1 — Supplementary Figure Legends [file 41420_2021_560_MOESM1_ESM.docx]

**Supplementary Results**

**Fig. S1. GO functional enrichment analysis on lncRNA associations.**

**Table S1. Primer sequences for quantitative real-time PCR.**

| **Primer names** | **Sequence (5'-3')** |
| --- | --- |
| CSN5-F | CACTGAAACCCGAGTAAATGC |
| CSN5-R | ACATCAATCCCAGAAAGCCAG |
| SNHG6-F | ATACTTCTGCTTCGTTACCT |
| SNHG6-R | CTCATTTTCATCATTTGCT |
| CTD-3065J16.9-F | TCCGGTTCCGGGGAACTAC |
| CTD-3065J16.9-R | CCAGGACAGGAAGTGACAGC |
| KB-1460A1.5-F | GACTTGAGAGTGTCCTCCGC |
| KB-1460A1.5-R | CTTCCATCGGGGCTACTGTG |
| CTD-3025N20.3-F | GTCTTCCGAACTCTGCTGCT |
| CTD-3025N20.3-R | TGTCACAGACGCACTCTTCC |
| RP11-295G20.2-F | GTGTGATCGTGGGAAAGCGA |
| RP11-295G20.2-R | TGCCAGAGCAGAACATGCCT |
| RP13-582O9.7-F | CTCTGGGCCTCCACATCTCG |
| RP13-582O9.7-R | TGGGAGTGTTCAGGTGTTTACTG |
| LINC01604-F | CAGAAGAGCTTCATCGGCCC |
| LINC01604-R | TCACAGACGCACTCTTCCCA |
| 18S-F | CAGCCACCCGAGATTGAGCA |
| 18S-R | TAGTAGCGACGGGCGGTGTG |

**Table S2. Clinical values of 7 lncRNAs and CSN5 in 35 HCC patients.**


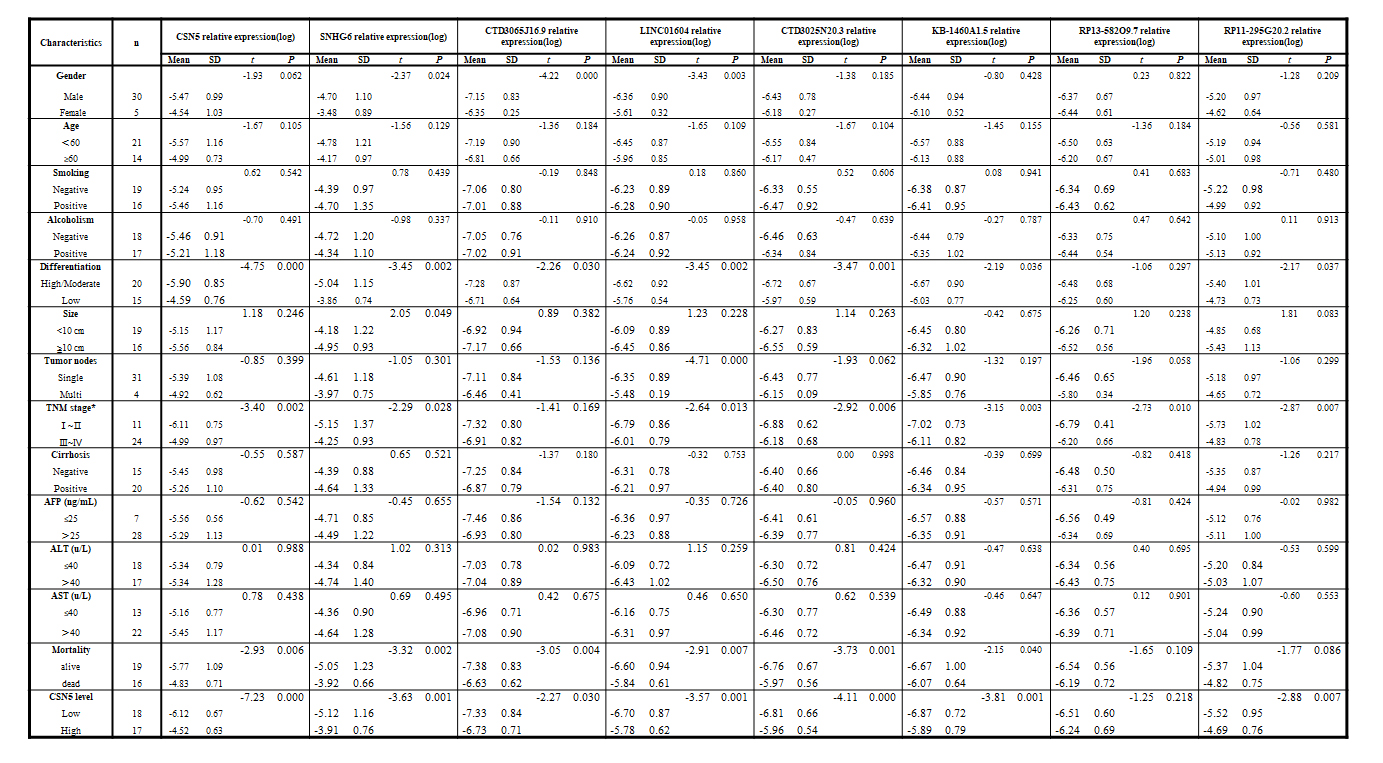


The student 𝑡-tests were applied to the analysis of data.
