# Supplementary figures and images for: Identification of a seven-long non-coding RNA signature associated with Jab1/CSN5 in predicting hepatocellular carcinoma
Source: Cell Death Discov. 2021 Jul 10;7:178. doi: 10.1038/s41420-021-00560-7 (PMC8272716; doi:10.1038/s41420-021-00560-7)

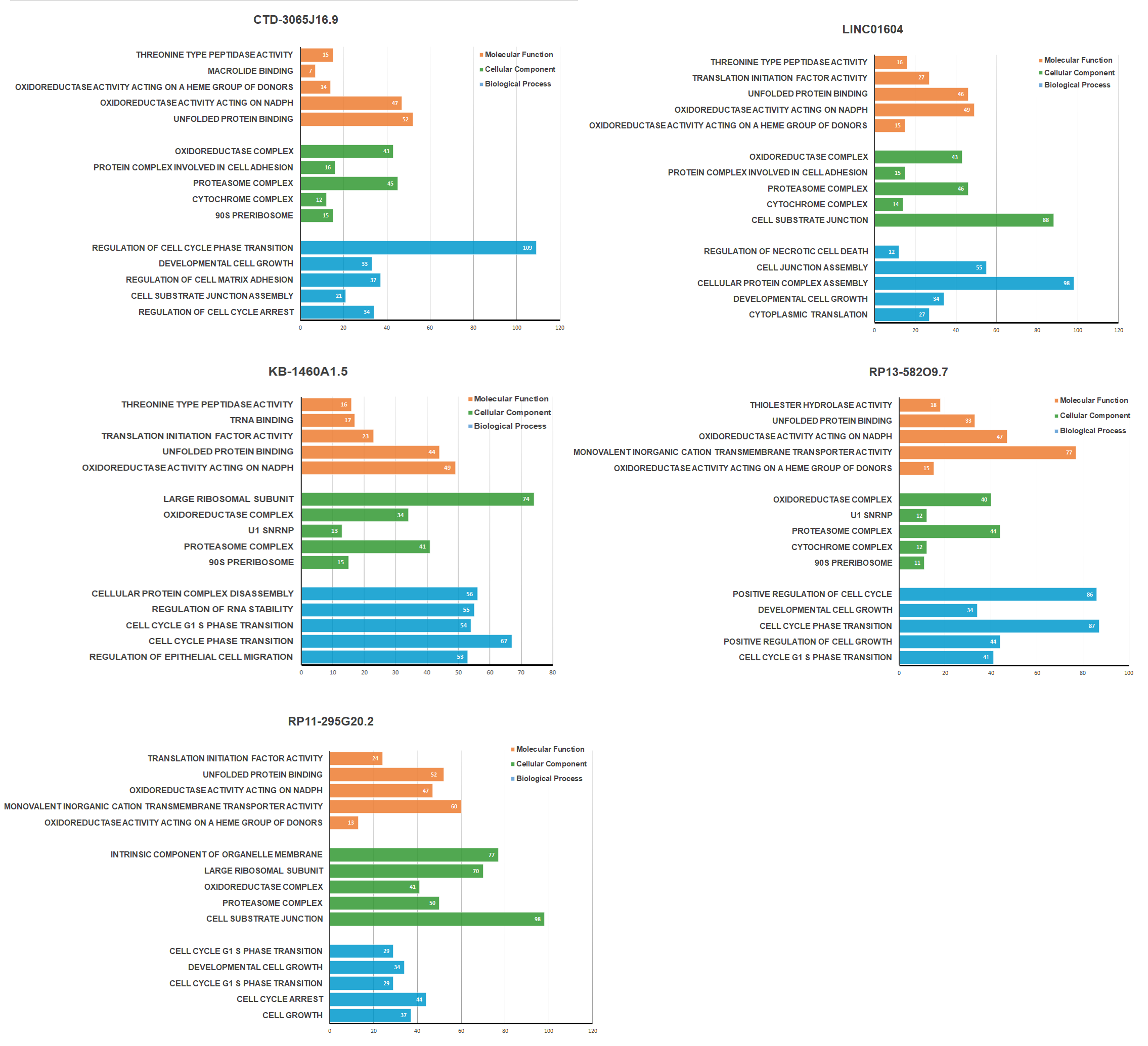

Supplement: Supplementary file 2 — Fig. S1 [file 41420_2021_560_MOESM2_ESM.tif]

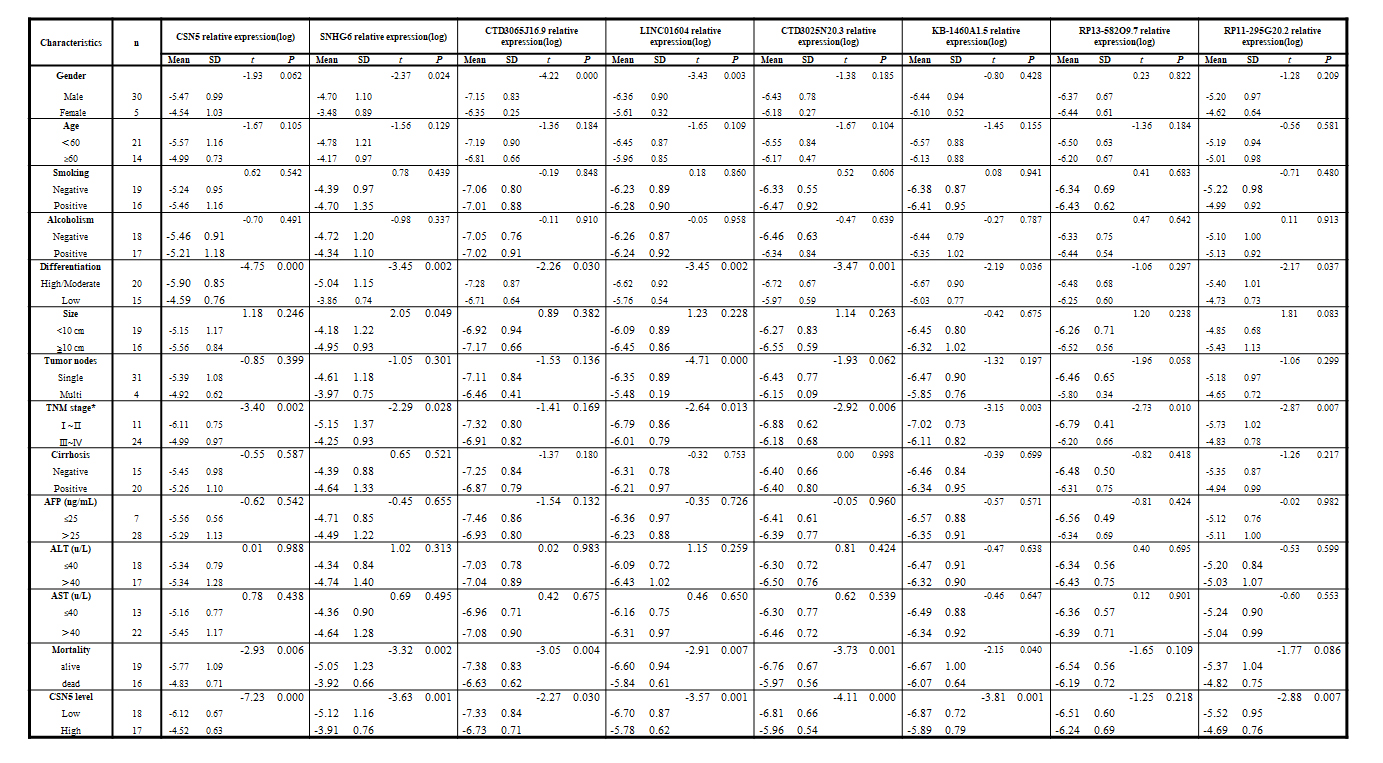

Supplement: Supplementary file 3 — Table-S2 [file 41420_2021_560_MOESM3_ESM.tif]
